# Supplementary figures and images for: Inferring the immune response from repertoire sequencing
Source: PLoS Comput Biol. 2020 Apr 29;16(4):e1007873. doi: 10.1371/journal.pcbi.1007873 (PMC7213749; doi:10.1371/journal.pcbi.1007873)

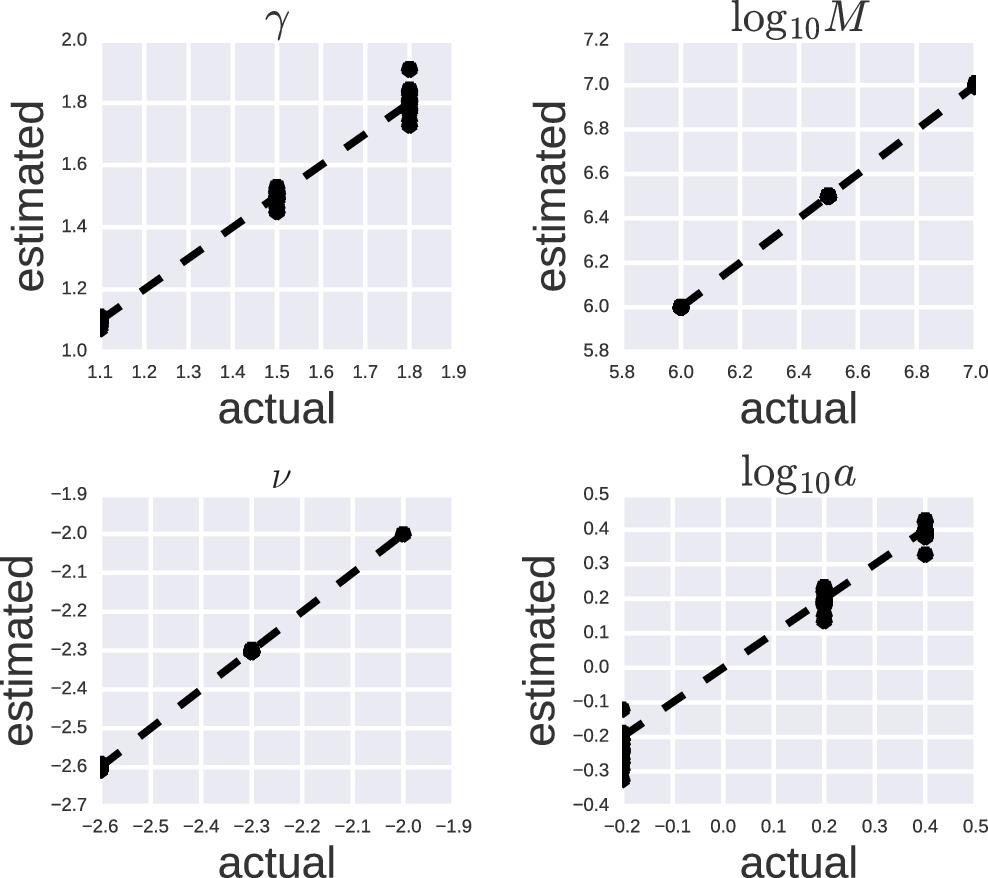

Supplement: S1 Fig — Shown are the actual and estimated values of the null model parameters used to validate the null model inference procedure over the range exhibited by the data. A 3x3x3x3 grid of points were sampled and results collapsed over each parameter axis. fmin was fixed to satisfy the normalization constraint. (TIF) [file pcbi.1007873.s001.tif]

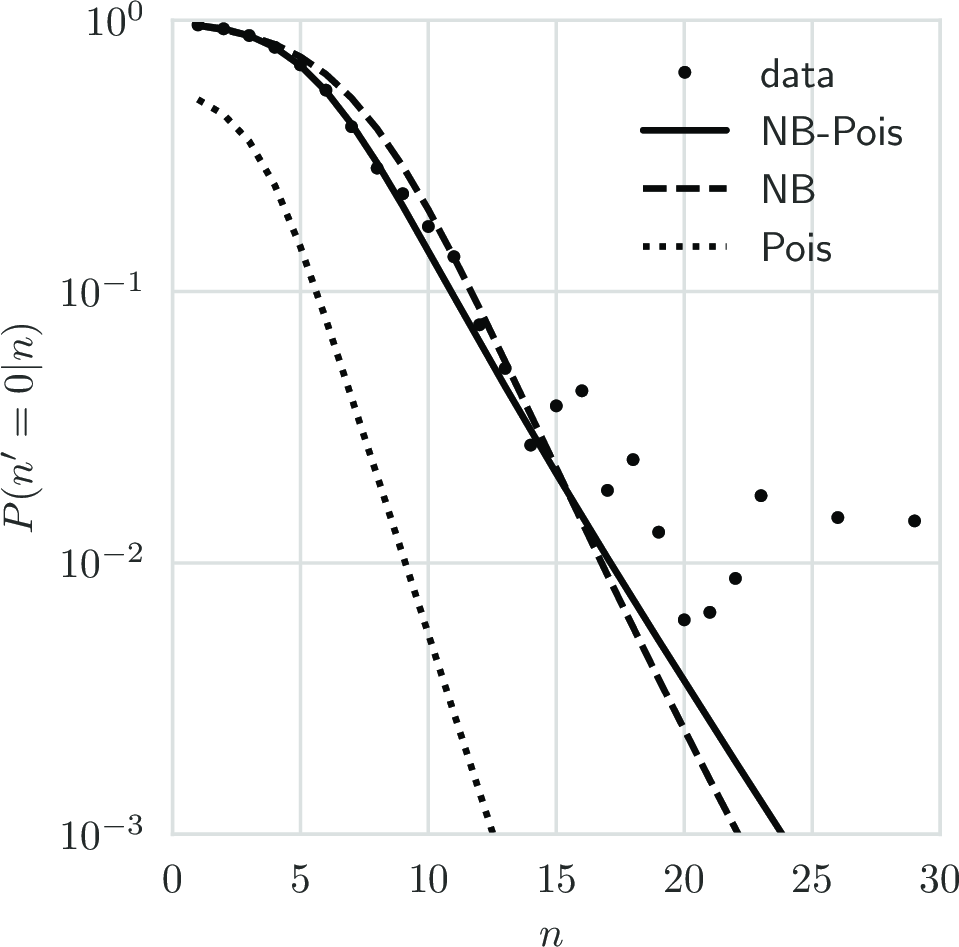

Supplement: S2 Fig — Two-step negative binomial to Poisson model captures tail better than one-step negative binomial model. Poisson model fits poorly. (Example donor S2-day 0 replicate pair). (TIF) [file pcbi.1007873.s002.tif]

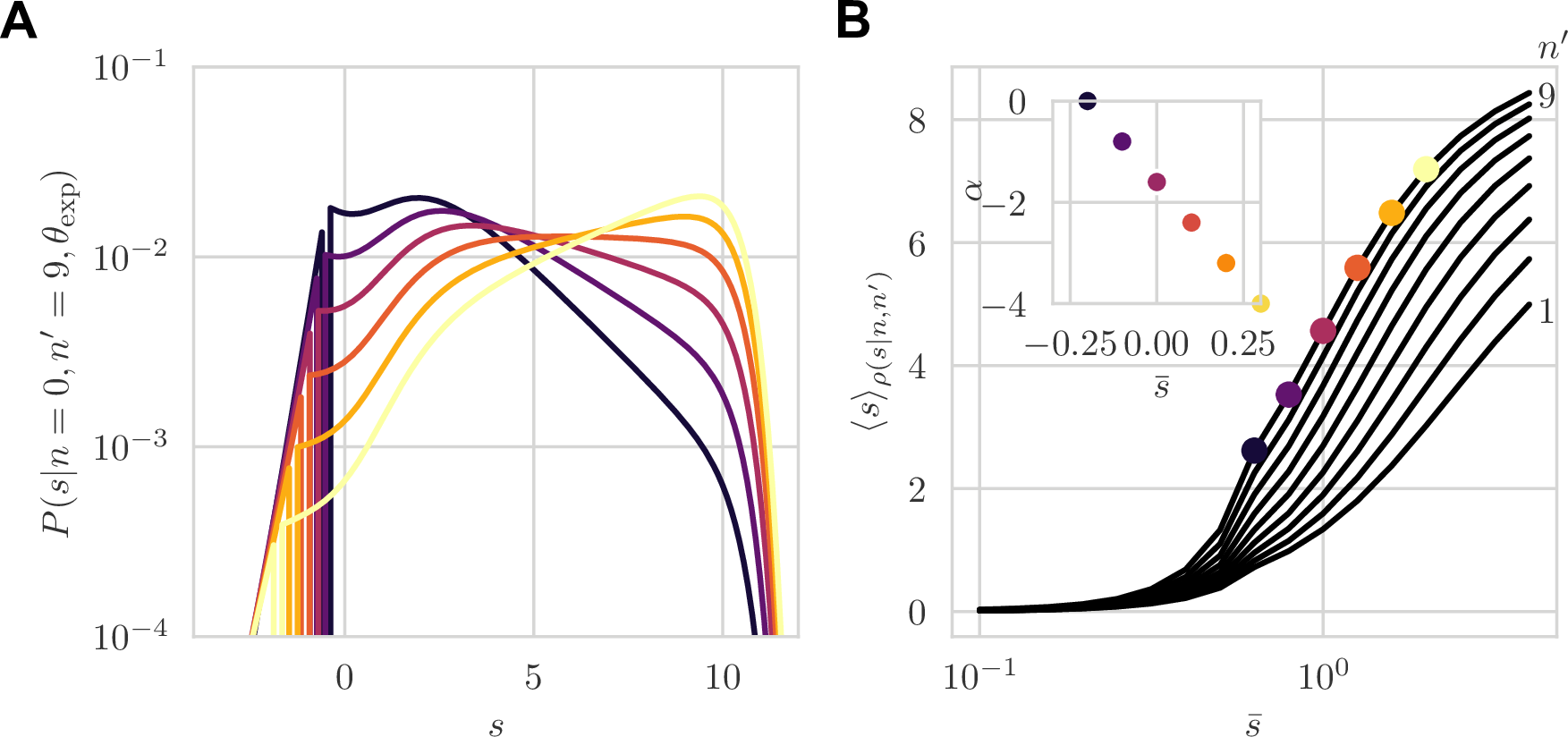

Supplement: S3 Fig — A) Posteriors for n′ = 9 over a range of (s¯,α) pairs spanning the ridge shown in the inset in (B) and Fig 7 along which the growth of s¯ leads to ρ(f) overwhelming ρs(s) as the dominant explanation for observed expansion. (B) The posterior mean versus s¯ for values of n′ = 1, …, 9, with the 5 values of s¯ used in (A) shown for n′ = 9. (TIF) [file pcbi.1007873.s003.tif]

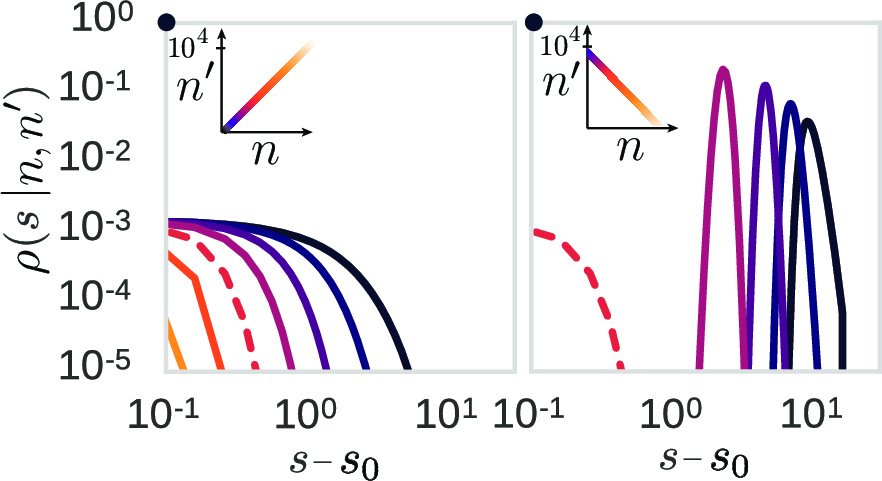

Supplement: S4 Fig — The black dot in both plots denotes the contribution of the non-responding component, ∝ δ(s − s0), to the posterior. (Parameters: N = 106, ϵ = 10−2). (TIF) [file pcbi.1007873.s004.tif]

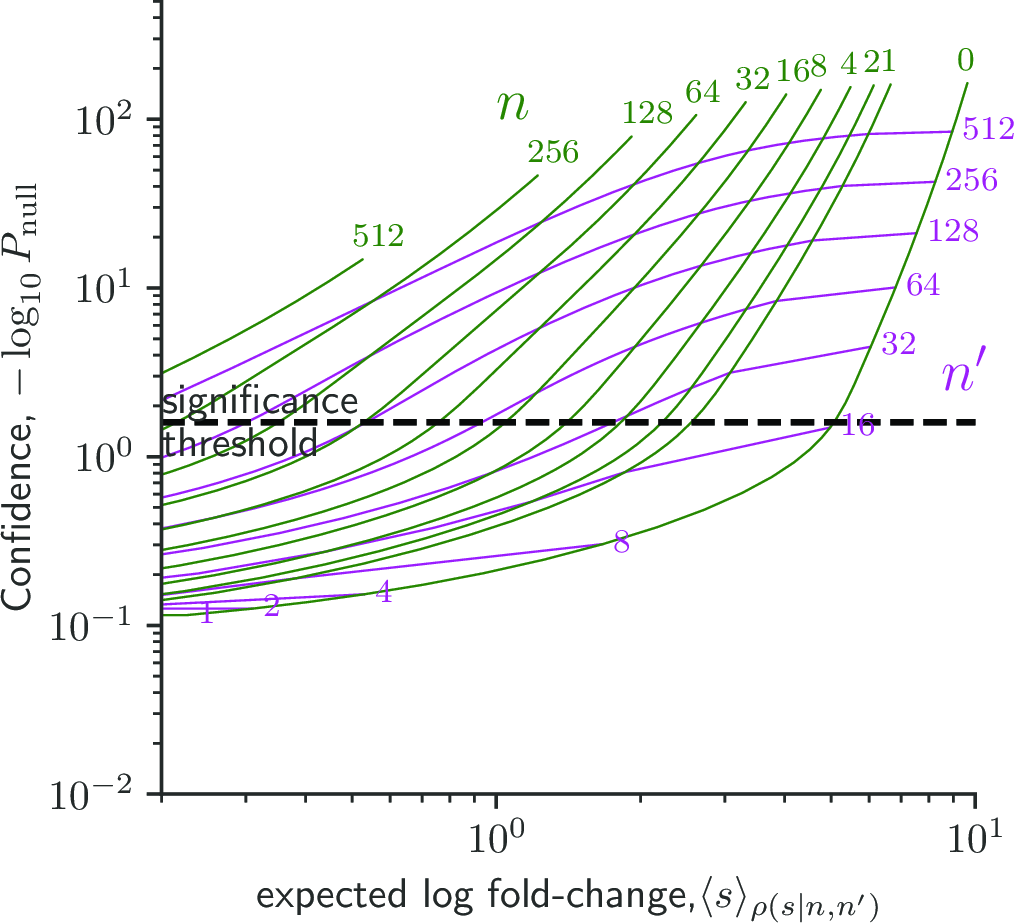

Supplement: S5 Fig — A significance threshold is placed according to Pnull = 0.025, where Pnull = P(s ≤ 0). (TIF) [file pcbi.1007873.s005.tif]
